# Improvement of vascular dysfunction by argirein through inhibiting endothelial cell apoptosis associated with ET-1/Nox4 signal pathway in diabetic rats

**DOI:** 10.1038/s41598-018-30386-w

**Published:** 2018-08-22

**Authors:** Jie Su, Xing-Rong An, Qing Li, Xiao-Xue Li, Xiao-dong Cong, Ming Xu

**Affiliations:** 1 Department of Clinical Pharmacy, School of Preclinical Medicine and Clinical Pharmacy, China Pharmaceutical University, Nanjing, 210009 China; 2 Department of Pathology, Medical School of Southeast University, Nanjing, 210009 China; 3 School of Pharmacy, Zhejiang Chinese Medical University, Hangzhou, 311400 China

## Abstract

Endothelial cell apoptosis plays an important role in the pathophysiological mechanism of vascular complications in type 2 diabetes mellitus (T2DM). Argirein, a new synthetic compound was demonstrated to inactivate NADPH oxidase to alleviate cardiac dysfunction in T2DM. Here, we investigated whether argirein medication attenuated the vascular dysfunction in T2DM by inhibiting endothelial cell apoptosis which was associated with NADPH oxidase. The rat aortic endothelial cells (RAECs) were incubated with glucose (30 mM) for 48 hour *in vitro*. It was shown that high glucose significantly increased the protein expression of BAX (Bcl-2 Associated X protein) and Caspase-3 and decreased Bcl2 (B-Cell Leukemia/Lymphoma 2) protein level in RAECs, which was normalized by argirein medication. The annexin V-FITC bound cell percentage and DNA fragments in agarose electrophoresis were markedly suppressed by argirein to confirm the anti-apoptotic property of argirein in RAECs. Furthermore, we found that argirein blocked the endothelin (ET)-1/Nox4 signal-dependent superoxide (O_2_^−.^) generation, which regulated endothelial cell apoptosis in RAECs. *In vivo*, argirein intervention relieved the vasodilatory response to acetylcholine and restored the expressions of Nox4 and BAX in the aorta endothelium of high-fat diet (HFD)-fed rats following streptozocin (STZ) injection. For the first time, we demonstrated that argirein could inhibit vascular endothelial cell apoptosis, which was attributed to blocking ET-1/Nox4 signal-dependent O_2_^−^ generation in RAECs. This current study revealed the therapeutic effects of argirein to prevent the vascular complication in T2DM through inhibiting endothelial cell apoptosis which was associated with the anti-oxidative property of argirein.

## Introduction

Type 2 diabetes mellitus (T2DM) affects a rapidly growing portion of the global population and is prone to an array of vascular complications^1^. Abnormal endothelial function is considered as early critical event in atherogenesis to contribute to clinical vascular lesion in diabetes^2^. Endothelial cell apoptosis may increase smooth muscle cell proliferation and migration, enhance blood coagulation and increase leukocyte infiltration into the endothelium thus leading to endothelial dysfunction^3^. Thus, novel therapeutic strategies that restore endothelial function by anti-apoptosis hold promise as interventions to lower cardiovascular risk in diabetes.

**Figure 1:**
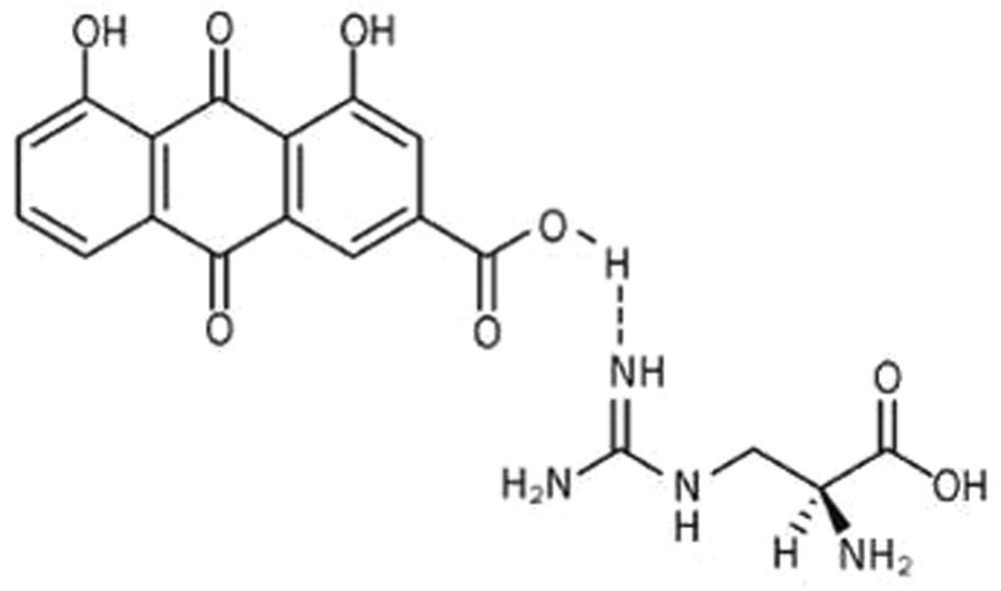
The chemical structure of argirein compound shows a hydrogen bond connecting the two moieties.

Argirein, a new molecule, is combined rhein with L-arginine by a hydrogen bond forming a loose link between the two molecules (Fig. 1)^4^. The chemical modification by connecting a moiety of L-arginine is conducted to improve the biological behaviors of rhein. Pharmacokinetic studies have shown that argirein is metabolized to release rhein and L-arginine with extended plasma clearance time, such as delayed C_max_, reduced T_max_ and longer T_1/2_^4^. Argirein manifests anti-oxidative activities of rhein and the NO offering activity of L-arginine. It was reported that argirein could substantially relieve isoproterenol-induced exacerbation of cardiac failure and alleviate cardiac dysfunction in T2DM, which was associated with suppressing NADPH oxidase activity^5,6^. The vasculature is a rich source of NADPH oxidase which produces most of the reactive oxygen species (ROS) and the toxic roles of ROS in endothelial apoptosis has been widely recognized and deeply investigated^7^. Additional study from our group found that argirein blunt hepatosteatosis in diabetic rats fed with a HFD for 12 weeks combined with a single low dose of STZ, which was manifested as inhibiting DNA ladder and upregulated Bcl2 and downregulated Bax in hepatocyte apoptosis^8^. Here, we investigated whether argirein medication attenuated vascular lesion and dysfunction in T2DM rats by inhibiting endothelial cell apoptosis which was associated with the anti-oxidative property of argirein.

Hyperglycemia-triggered endothelial cell apoptosis is a critical event in the process of diabetes-associated vascular disease^9^. Thus, 30 mM glucose was used to induce the apoptosis in RAECs which was assessed by western blotting assay, flow cytometer and DNA ladder. *In vivo* study, vascular dysfunction was produced in T2DM rats fed with HFD combined with low dose STZ administration^10^. Nox4-dependent ROS aggregation is the main cause of vascular endothelial cell apoptosis^7^. Moreover, ET-1 is able to activate NADPH oxidase in endothelial cells resulting in the disturbance of vascular redox balance^11,12^. Considering the effects of argirein on attenuating ET-1/NADPH oxidase signal in renal tissue and myocardium^5,13^, we detected that the ET-1 system-dependent Nox4-dependent O_2_^−.^ was involved in the improvement of apoptosis by argirein intervention.

## Materials and Methods

### Isolation and culture of rat aortic endothelial cells

The thoracic aortas were separated from the male Sprague-Dawley(SD) rats, 250 ± 20 g, and immersed in 20% fetal bovine serum(FBS)-DMEM containing 1% antibiotics. The fat and connecting tissue were rapidly removed with fine tweezers under a stereoscopic microscope. By using fine needles, the thoracic aortas were turned inside out, so that the endarterium could stay outward. Two sides of the vessel were bound and the turned-out endarterium were filled with collagenase type II solution (4 mg/ml, dissolved in serum-free DMEM). After the incubation for 45 min at 37 °C, RAECs were eluted from the thoracic aortic vessel by flushing with 5 ml of DMEM containing 20% FBS. RAECs were collected by centrifugation at 1,000 rpm for 5 min. Then the precipitate was gently resuspended by pipette with 2 ml of heparin (90 U/ml), endothelial growth factor (EGF, 10ug/mg) and 20% FBS-DMEM which was cultured in a gelatin-coated culture dishes. The medium was replaced twice each week until the cells grew to 80–90% confluence, the passage number of exponential phase cells was about 4~6 times.

**Figure 2:**
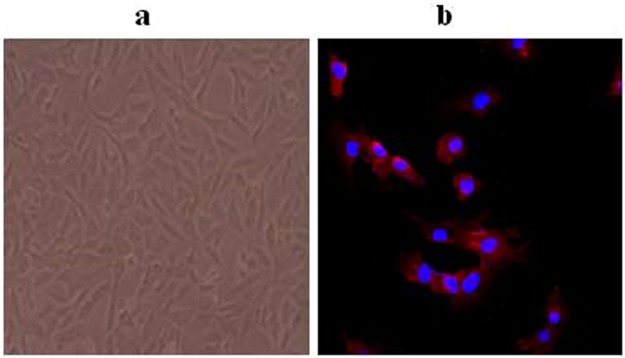
Primary endothelial cell characterization. **(a)** Cell morphology of RAECs under light microscopy showed the typical two-demensional cobblestone. **(b)** Immunocytochemistry of RAECs that was stained with anti-vWF antibody followed by a dylight 594-conjugated second antibody, and photographs were at 400x magnification (n = 4).

RAECs were identified according to their morphology and immunocytochemical staining of marker protein. RAECs were arrayed into a cobblestone-like structure and formed a confluent layer of endothelial cells after 3~4 days culture (Fig. 2a)^14^. As shown in Fig. 2b, von willebrand factor (vWF), a well-recognized endothelial cell marker followed by a dylight 594-conjugated second antibody was used to identify RAECs^15^.

### Western blotting analysis

The protein expression of BAX, Bcl-2, Caspase-3, Nox4, and ET_A_R were analyzed by western blot which was performed as we described previously^16^. In brief, RAECs were washed twice with phosphate-buffered saline (PBS) and scraped in radio immunoprecipitation assay (RIPA) lysis buffer (BU Technology CO, LTD) for 15 min at 4 °C. Cell lysate was centrifuged at 10,000 rpm/min at 4 °C for 10 min, and the supernatant was collected and determined by BCA protein quantitative kit (BU). After boiling for 5 min at 95 °C in a 5 × loading buffer (BU Technology CO, LTD), 30 μg protein was separated by 10% SDS-PAGE at 80 V for 30 min and 120 V for 1 h. Then protein was electrophoretically blotted to polyvinylidene fluoride membranes (PVDF membrane, 0.45 μm, Millipore Co. Ltd.) at 200 mA for 1 h. The membranes were blocked with 5% non-fat milk (5% w/v) in Tris-buffered saline with Tween 20 for 1.5 h. After washing three times with TBST, the membranes were probed with primary antibodies of BAX (1:500, Proteintech), Bcl-2(1:1000, CST), Caspase-3(1:500, Wanleibio), Nox4 (1:1000, Abways), and ET_A_R (1:1000, Sigma) overnight at 4 °C. β-actin(1:5000, Abways) or β-tubulin(1:5000, Abways) were used as control protein. After three times of wash with TBST, the membranes were incubated with the goat anti-Rabbit IgG(1:5000, Abways) as secondary antibody at room temperature for 1.5 h. The blot was detected by chemiluminescent detection systems with Super ECL Plus Detection Reagent (1:1, BU). Densitometric analysis of the images was performed with ImageJ software (NIH, Littleton, CO, USA).

### Apoptosis assay by flow cytometry

The apoptosis of RAECs was detected using an Annexin V-FITC Apoptosis Detection Kit (Vazyme Biotech) according to the manufacturer’s instructions. RAECs (1 × 10^5^ cells/well) were trypsinized and centrifugated at 1000r/min for 5 min at 4 °C. The cells were washed twice in cold (4 °C) PBS and then subjected to another centrifugation, and then the cells were resuspended in 100 µL Binding Buffer (5 × 10^5^ cells/100 µL). After the incubation with 5 µL Annexin V-FITC and 5 µL propidium Iodide (PI) staining solution for 10 min without light, the cells were detected by a flow cytometer (MACSQuant) within 1 hour. The data was analyzed by FlowJo 7.6 software, and the percentage of cells to total cells was calculated as the rate of apoptotic cells.

### DNA ladder assay

Fragmented DNA was a biochemical hallmark of apoptosis characterized by ladder DNA which was extracted by using the apoptotic DNA ladder kit (Nanjing Angle Gene Biotechnology co. LTD). DNA extraction from RAECs was conducted according to the manufacturer’s instructions. DNA extraction was treated with RNase for 15 min at room temperature and proteinase K. DNA samples were applied to a 1% agarose gel electrophoresis at 60 V for 3.5 h.

### TUNEL assay

A high sensitive one-step Terminal deoxyribonucleotide transferase dUTP nick end labelling (TUNEL) apoptosis assay kit (Beyotime Institute of Biotechnology, Jiangsu, China) was used to label DNA strand break according to the manufacturer’s instruction. TUNEL reagent was performed on RAECs and rat thoracic aortas to detect apoptosis. RAECs were fixed with 4% paraformaldehyde for 30 min. And after washing with PBS, they were permeabilized by 0.3% Triton X-100. For tissue slides, paraffin sections were firstly dewaxed with dimethylbenzene and rehydrated with ethanol solution (gradient concentration) and distilled water. Then cells and slides share the same step. Sections were immersed in 50 μl TUNEL reaction fluid in a humid environment at 37 °C for 1 h. After washing twice, sections were incubated with 4′, 6-diamidino-2-phenylindole (DAPI) to stain nuclei. Finally, samples were observed under fluorescence microscope.

### Measurement of O_2_^-^ production

Total O_2_^−.^ generation by RAECs was measured using DHE (Sigma, CAS 104821-25-2). DHE was oxidized by intracellular O_2_^−.^ and then incorporated into the chromosomal DNA in the nucleus to produce red fluorescence. After treatment, RAECs (1 × 10^4^cells/well) in 6-well plates was washed twice with PBS. Then cells were incubated with 10 μM DHE diluted in 2 ml serum free-RPMI 1640 without light at 37 °C. After 30 min incubation, RAECs were washed three times with 1–2 mL PBS, red fluorescent images were observed by fluorescence microscopy. The fluorescence intensity was analyzed by ImageJ software and the ratio of fluorescent intensity to that at basal level was quantified to represent cellular O_2_^−.^ level.

### RNA interference of Nox4

Nox4 siRNA was purchased from Santa Cruz (sc-61887). Their targeting sequences were as follows: 5′-AAUUCUCCGAACGUGUCACGU-3′. The scrambled small RNA (5′-AAUUCUCCGAACGUGUCACGU-3′) was confirmed as non-silencing double stranded RNA and was used as control in current study. Transfection of siRNA and scramble RNA were performed using the GeneTran™ III (BIOMIGA) according to the manufacturer’s instructions. The siRNA of Nox4 was confirmed with western blot analysis.

### ESR spectrometric detection of O_2_^−^

For the detection of the O_2_^−.^ production dependent on NADPH oxidase, the proteins extracted from RAECs using sucrose buffer were resuspended with modified Krebs-Hepes buffer containing deferoximine (25 μM, Sigma) and diethyldithiocarbamate (5 μM, Sigma). 10 μL protein samples (1 μg/μL) were immediately mixed with 80 μL KHB buffer, followed by 1 mM of the cell permeable O_2_^−.^ specific spin trap, 1-hydroxy-3-methoxycarbonyl- 2,2,5,5- tetramethylpyrrolidine (CMH; ENZO, Alexis Corporation), and substrate NADPH (20 μM, Beyotime Biotechnology) in the presence or absence of manganese-dependent superoxide dismutase (SOD, 8000U/ml; Beyotime Biotechnology). The mixture was loaded into glass capillaries and immediately analyzed for O_2_^−.^ production kinetically for 10 min using an ESR spectrometer (Germany BRUKER) as we previously described^17^. The SOD-inhibitable fraction of the signal in the homogenates reflected the total O_2_^−.^ level, and the results were expressed as fold changes relative to control.

### Establishing of type 2 diabetic rat model

Adult male SD rats, weighing 230–250 g, were purchased from Qinglongshan Lab Animal Ltd, Nanjing, China. Animal handling and experimental procedures were approved by the ethic committee of China Pharmaceutical University, in accordance with the Guidelines of Animal Experiment set by the Bureau of Sciences and Techniques of Jiangsu Province, China [NO.SYXK2007–0025].

SD rats were developed as a rat model of T2DM according to the method in our previous study^18^. Rats were fed with a HFD (22 g/d) consisting of 10% saccharose, 10% lard, 10% sugar, 5% egg yolk powder, 0.5% cholesterol, 74.5% basal chow. The ordinary chow was constituted by 36% corn, 23% triturate wheat, 10% bran, 12% soy bean powder, 3% egg; 12% fish powder, 2% driedyeast, 1% of amixture of calcium bicarbonate, multi-vitamins and micro-elements. Both the normal chow and high-fat diet were purchased from Qinglongshan Lab Animal Ltd (Nanjing, China).

Rats were divided into three groups (n = 8): control, untreated diabetes, and diabetes treated with argirein(200 mg/kg, i.g.). In the first 6 weeks, rats in untreated and treated diabetes group were given a HFD alone and rats in the control group only received regular chow. On the beginning of 7th week, rats fed with a HFD received a single time intraperitoneal injection of STZ (40 mg/kg, dissolved in pH 4.5 citrate buffer). Control rats only received an equivalent volume of citrate buffer. After the injection of STZ, all groups maintained their original diets. Rats on HFD with low STZ were recognized as diabetic while the FBG level reached 16.7 mM after 1 week of STZ injection. The intervention with argirein (200 mg/kg/day, i.g.) was conducted once a day for last 3 weeks. However, rats in the groups of normal and untreated diabetes were given an equal volume of saline solution. A gain of body weight was monitored at an interval of 3 days.

Blood samples from the rats were collected and centrifuged at 3000 g for 10 min at 4 °C.The serum was collected and stored at −20 °C before use. FBG and fasting insulin (FIN) were assayed following instructions of the kits provided by the Nanjing Jiancheng Bio engineering Institute (China).

### Immunohistochemistry

The aortic rings were dissected and perfused with PBS and fixed in 4% paraformaldehyde (PFA) and embedded in paraffin. The paraffin sections of the aortic rings were sliced, dewaxed and washed with PBS three times for 5 min, followed by blocked with 3% H_2_O_2_ for 10 min and 5% BSA for 1 hour at room temperature. For immunohistochemical studies, the sections were incubated with anti-Nox4 (1:100, Abways, China) and anti-BAX (1:50, Proteintech) at 4 °C overnight. After washing three times with PBS for 5 min, the sections were incubated with the rabbit anti-goat biotinylated secondary antibody (dilution 1:100, Abways) for 2 hours at room temperature. Diaminobenzidine (DAB) chromogen kit (Beyotime Biotechnology co, LTD.) was used for staining. The sections were counterstained with hematoxylin before examination under OLYMPUS CX31 microscope.

### Vascular tension recording of rat thoracic aorta

Rats were anaesthetized by 3% chloral hydrate, then thoracic aorta was quickly removed and immersed into ice-cold Krebs Henseleit solution (mM) (KH, pH 7.4, 119.0 NaCl, 25.0 NaHCO_3_, 11.1 Glucose, 2.4 CaCl_2_, 4.7 KCl, 1.2 KH_2_PO_4_, 1.2 MgSO_4_, 0.024 Na_2_EDTA) solution. The aortas were subtracted from free of connective tissue and fat, and then cut into three separated thoracic aortic rings with a width of approximately 2 mm. All anatomical procedures were carefully done in order to protect the endothelium from accidental damage. Aortic rings were then suspended in water-jacketed tissue baths and tested for isometric force recorded by a PowerLab system. The KH solution maintained at 37 °C and the mixed gas contained 95% O_2_ and 5% CO_2_ was continuously bubbled through the bath. The baseline tension placed on the aortic rings was adjusted to 2 g. The isometric tensions were recorded using a force displacement transducer (Chengdu TaiMeng).

After three equilibration, the aortic rings were contracted with phenylephrine (1 μM) to obtain a maximal response, and then a cumulative dose-response curve to acetylcholine (1 × 10^−9^–10^−4^M) and SNP (1 × 10^−9^–10^−4^M) was determined in thoracic aorta, respectively. *In vitro*, the aorta rings were pre-incubated with 50 mM glucose for 3.5 h to induce the endothelial vasodilation dysfunction. Nox4 inhibitor GKT137831 (10^−5^M) was incubated with aorta rings for 0.5 h.

### Statistical analysis

Data are presented as the mean ± SE. Significant differences between and within multiple groups were examined using analysis of variance for repeated measures, followed by Duncan’s multiple-range test. Student’s *t* test was used to detect significant differences between two groups. *p* < 0.05 was considered statistically significant.

## Results

### Argirein inhibited the apoptosis of RAECs under high glucose

**Figure 3:**
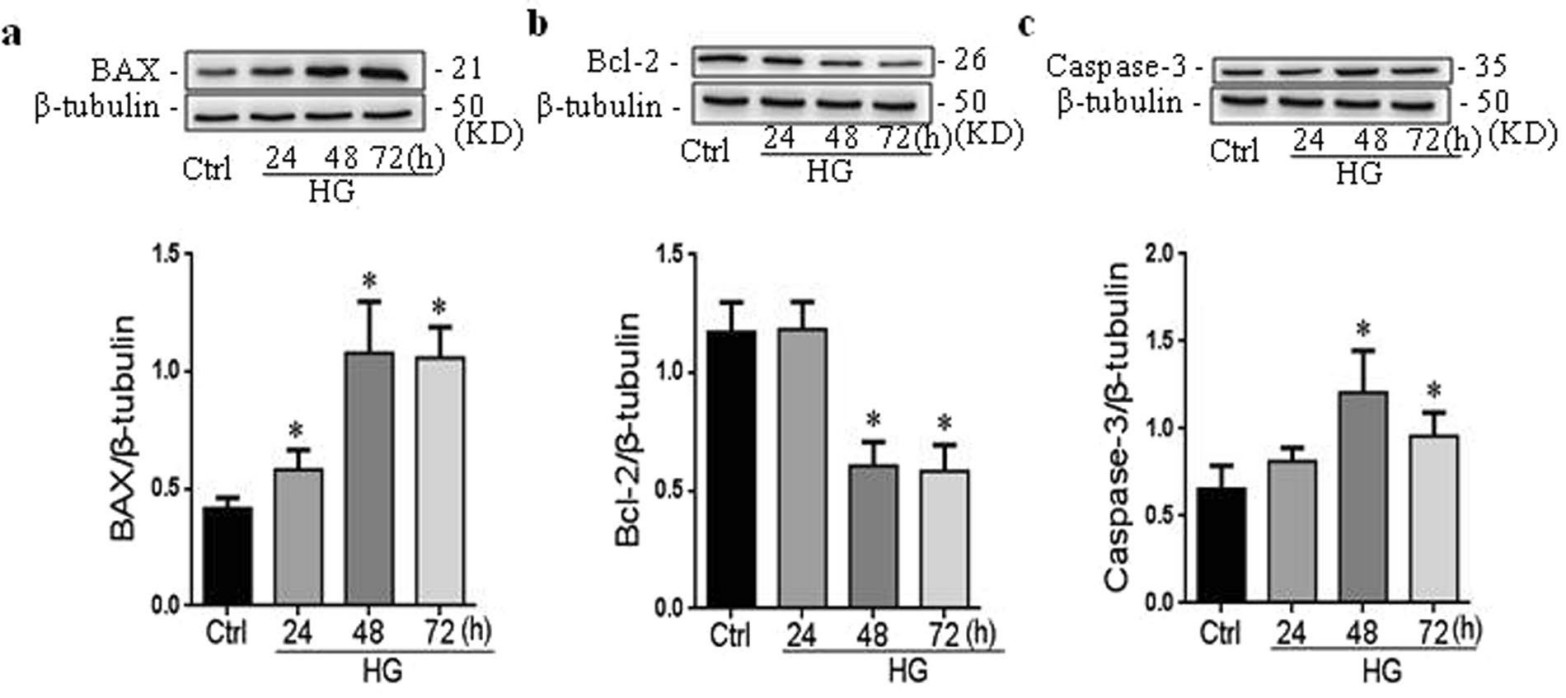
High glucose induced apoptosis-related protein expression in different time. RAECs were incubated with glucose at the dose of 30 mM for 24, 48, 72 hours. Representative western blot gel documents and summarized data showing the protein expression of BAX, Bcl-2 and caspase-3 **(a–c)**. The original gel documents of western blot were shown in Fig. 3 in Supplementary Information. ^*^P < 0.05 *vs*. Control (Ctrl) (n = 4).

**Figure 4:**
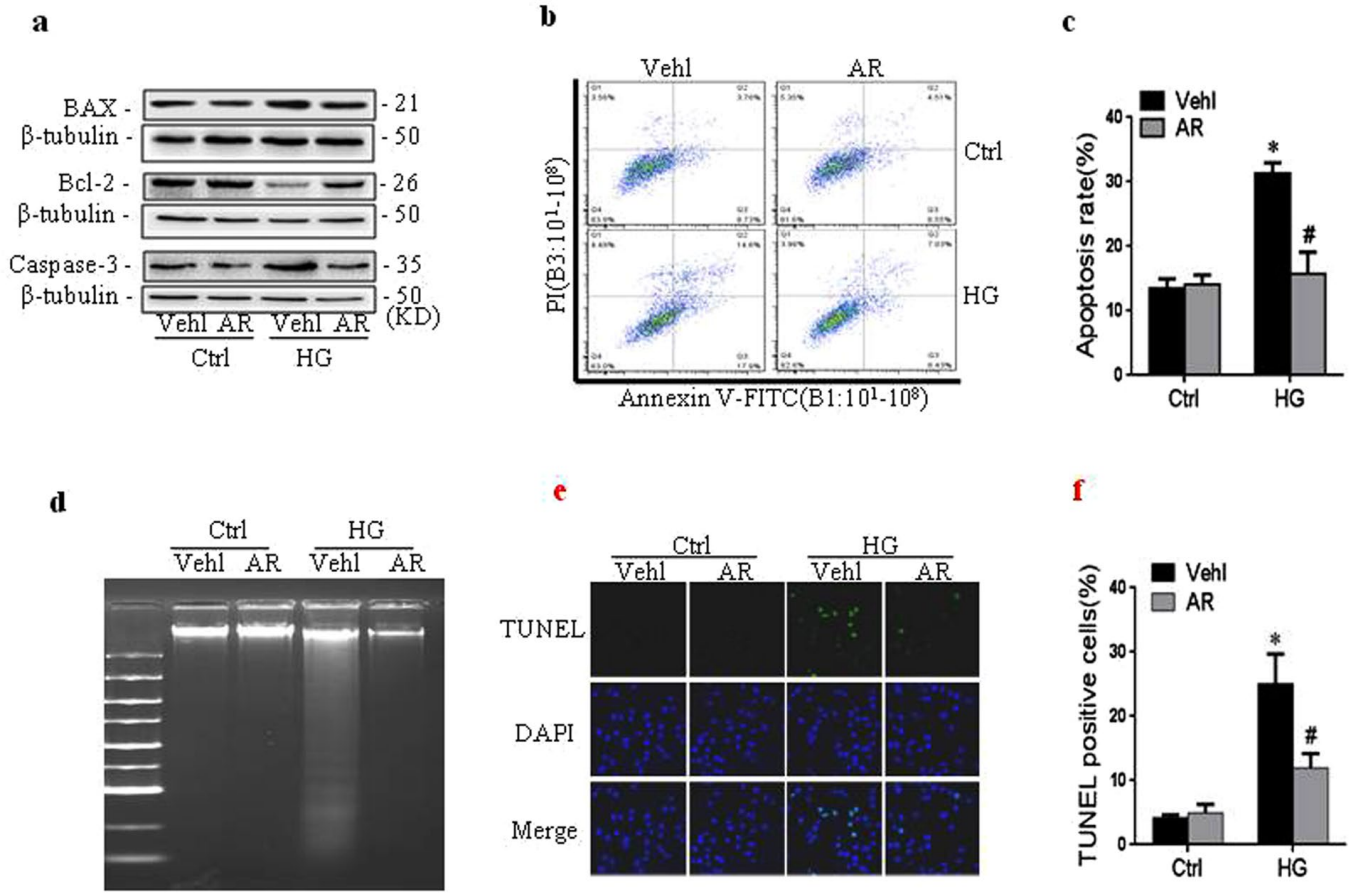
Argirein inhibited the apoptosis of RAECs under high glucose. RAECs were incubated with high glucose (HG, 30 mM) for 48 h in the presence or absence of argirein (AR, 1 μM) treatment for the last 12 h. Representative western blot gel documents (**a** and Fig. 4a in Supplementary Information.) showing the protein expression of BAX, Bcl-2 and Caspase-3. Representative plots **(b)** and summarized data **(c)** showing the apoptotic populations of RAECs (the bottom right of the plots) sorted by flow cytometry after they were double-stained with FITC-annexin V and propidium iodide (PI). **(d)** DNA ladders showing DNA fragments in 1% agarose gel electrophoresis. Representative fluorescent images of TUNEL assay **(e)** and summarized data **(f)** showing the TUNEL positive cells. Photographs were at 400x magnification. *P < 0.05 *vs*. Vehicle (Vehl) Ctrl; ^#^*P* < 0.05 *vs*. HG alone treated group (n = 4).

The members of the B cell lymphoma 2 gene families, such as BAX and Bcl-2, have a central role in regulating programmed cell death by controlling pro-apoptotic and anti-apoptotic intracellular signals in many cell types, and the activation of the effectors caspase-3 mediates the apoptosis. In order to optimize the condition of high glucose-induced apoptosis, RAECs were incubated with glucose at the dose of 30 mM for 24, 48, 72 hours. It was found that the most remarkable change of BAX, Bcl-2 and caspase-3 responded to high glucose for 48 hour (Fig. 3a–c). Thus, 30 mM glucose with the incubation for 48 h was used as an optimal condition to trigger apoptosis *in vitro* study. After the argirein intervention, the protein levels of BAX and Caspase-3 decreased compared with control (Fig. 4a). On the contrary, the expression of Bcl-2 protein significantly increased in response to argirein (Fig. 4a).

Annexin V-FITC/PI double staining was further used to investigate anti-apoptotic role of argirein by flow cytometric assay. The results manifested that the Annexin V-FITC bound cell percentage of RAECs increased from 12.49% in control group to 32.50% in high glucose-treated group, while argirein intervention significantly inhibited the total apoptosis ratio to 13.46% (Fig. 4b,c). In agarose electrophoresis, DNA fragments serves as an important symbol to reflect cell apoptosis by apoptotic DNA ladder kit^8^. As shown in Fig. 4d, high glucose treated group exhibited clear DNA ladder, which was dramatically improved by argirein intervention. Apoptotic cells labeled with TUNEL staining showed a cytoplasmic fluorescence which distinguished them with normal cells and necrotic cells. After TUNEL labeling, the TUNEL-positive cells were very low in normal control by fluorescence microscope. In contrast, high glucose-treated RAECs showed more TUNEL-positive cells to certify increased apoptotic ratio, which was significantly attenuated by the pretreatment with argirein (Fig. 4e,f). These assay above further consistently confirmed the anti-apoptotic role of argirein on in RAECs under high-glucose condition.

### Argirein decreased O_2_^−.^ production under high glucose

**Figure 5:**
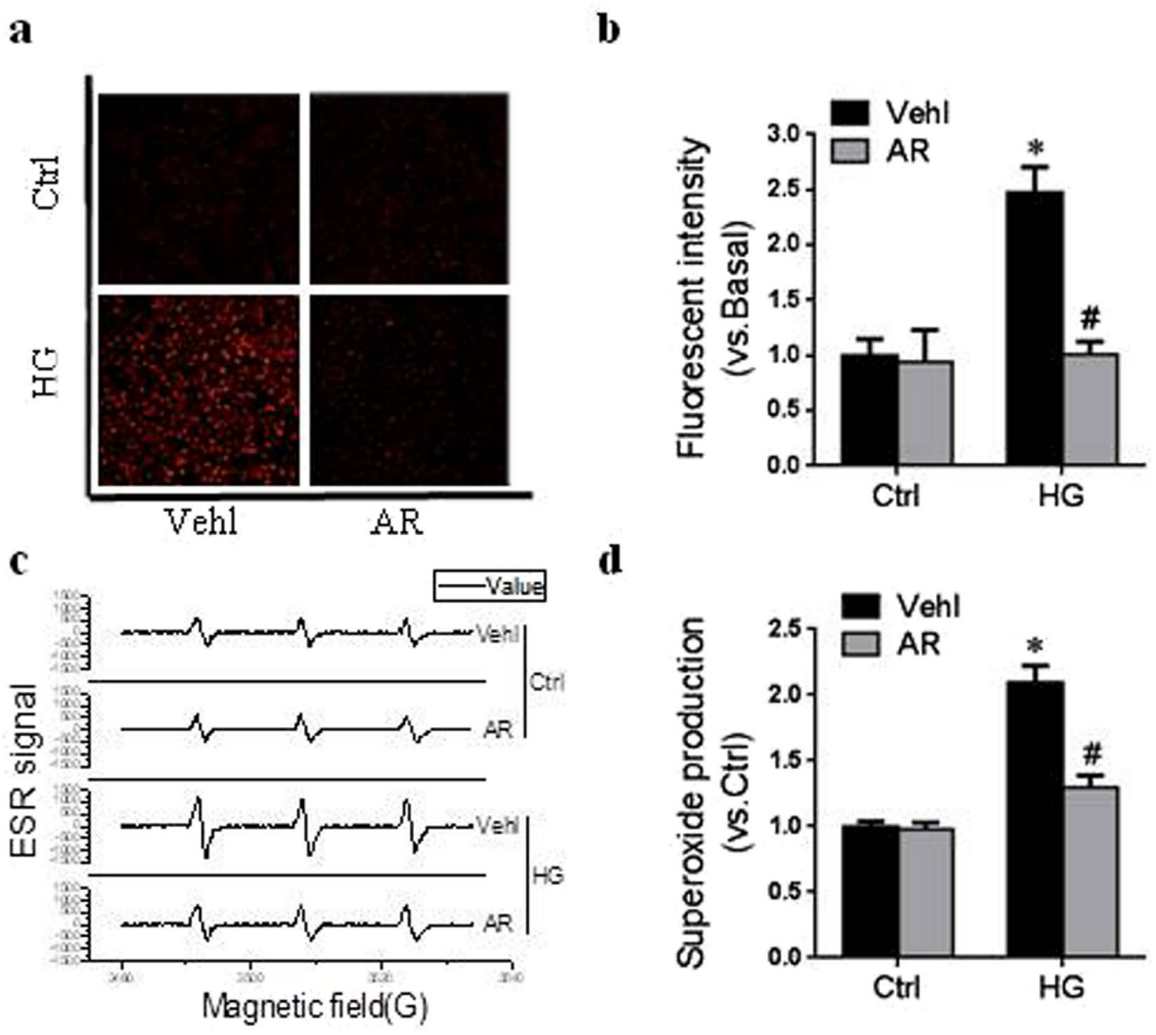
Argirein decreased O_2_^−.^ production under high glucose. Typical representative fluorescent images for DHE staining **(a)** and summarized data **(b)** showing the roles of argirein (AR, 1 μM) and on O_2_^−.^ production in RAECs incubated with high glucose (HG, 30 mM), and photographs were at 100 × magnification. Representative ESR spectrographs of O_2_^−.^ trapped by CMH **(c)** and summarized data **(d)** showing O_2_^−.^ production dependent on NADPH oxidase in the RAECs. *P < 0.05 *vs*. Vehl Ctrl; ^#^*P* < 0.05 *vs*. HG alone treated group (n = 4).

Given the anti-oxidative prosperity of argirein, the fluorescent spectrometry of a fluorescent probe dihydroethidium (DHE) was employed to detect intracellular O_2_^−.^ production. RAECs treated with high glucose showed a strong red fluorescence produced from DHE oxidization by O_2_^−.^ (Fig. 5a). Argirein intervention apparently blocked the increase of fluorescent intensity in respond to high glucose (Fig. 5b). By using the eletron spin resonance (ESR) spectrometry, we further determined the role of argirein on high glucose-induced the O_2_^−.^ production in RAECs, Fig. 5c showed representative changes in superoxide dismutase (SOD)-inhibitable ESR spectrometric curve recorded by ESR spectrometer. The summarized data in Fig. 5d showed that high glucose significantly increased O_2_^−.^ production dependent on NADPH oxidase in the RAECs, which was markedly attenuated by argirein intervention. Taken together, these assays illustrated that argirein indeed had an anti-oxidative prosperity through suppressing NADPH oxidase-dependent O_2_^.-^ production.

### Nox4 mediated the anti-apoptotic role of argirein

**Figure 6:**
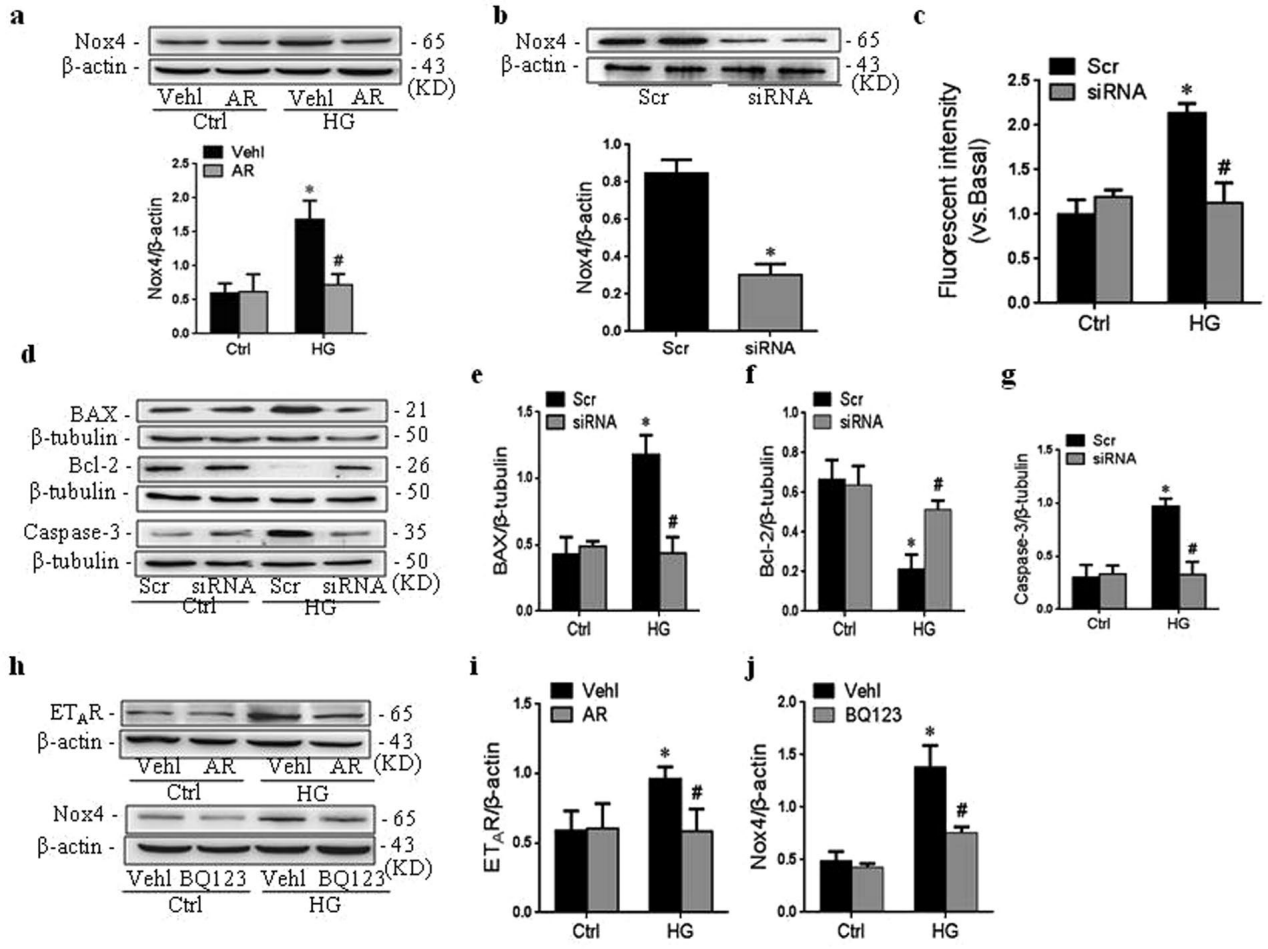
Nox4 mediated anti-apoptotic role of argirein. **(a)** Representative western blot gel documents and summarized data showing the roles of argirein (AR, 1 μM) on Nox4 protein expression. After Nox4 siRNA transfection, Nox4 protein expression **(b)** and O_2_^−.^ production with DHE staining **(c)** and the apoptotic-related protein expression including BAX, Bcl-2, and Caspase-3 **(d–g)** in RAECs incubated with high glucose (HG, 30 mM). Representative western blot gel documents and summarized data showing the roles of AR on ET_A_R protein expression **(h**,**i)** and the roles of specific ET_A_R inhibitor BQ123 on Nox4 protein expression **(h**,**j)** in RAECs. The original gel documents of western blot were shown in Fig. 6a,b,d,h in Supplementary Information. *P < 0.05 *vs*. Vehl or Scramble (Scr) Ctrl; ^#^*P* < 0.05 *vs*. HG alone treated group (n = 4).

In order to dive deeper into the molecular mechanisms underlying the role of argirein in O_2_^.-^ production, the possible involvement of Nox4 was examined. Here, we found that Nox4 protein dramatically increased in RAECs incubated with high glucose, which was inhibited by argirein (Fig. 6a). However, there was no significant difference in Nox1 or Nox2 protein expression after the treatment with argirein (not shown). To elucidate the role of Nox4 protein on apoptosis in RAECs, we next examined whether RAECs lacking Nox4 gene exhibited the apoptosis. Nox4 siRNA was transfected into RAECs and the apoptosis was observed, which efficacy of Nox4 siRNA transfection in RAECs was 64.1% (Fig. 6b). The apparent inhibition of O_2_^.-^ production due to Nox4 siRNA transfection confirmed the transfection efficacy Nox4 siRNA (Fig. 6c), suggesting that O_2_^.-^ production during high glucose stimulation was mainly originated from Nox4 activation. As showed in Fig. 6d–g, Nox4 siRNA resulted in a significant decrease in the level of Bax and caspase-3 as well as the increase of Bcl-2 under high glucose stimulation.

ET-1 mediates Nox4-dependent oxidative stress through the combination with endothelin A receptor (ET_A_R) and/or endothelin A receptor (ET_B_R) *in vitro*^16^. As showed in Fig. 6h and i, the protein level of ET_A_R dramatically increased in RAECs incubated with high glucose, and argirein effectively refrained ET_A_R expression. Although ET_B_R was also upregulated by high glucose, there was no significant response to argirein medication (not shown). Furthermore, the pretreatment with ET_A_R antagonist BQ123 suppressed the protein expression of Nox4 in RAECs (Fig. 6h,j), which convincingly proved that ET_A_R indeed mediated the regulation of argirein on Nox4 expression. Taken together, these results indicated that the deactivation of ET_A_R/Nox4 signal pathway contributed to the inhibition of apoptosis by argirein.

### Argirein attenuated vascular dysfunction in T2DM rats

**Figure 7:**
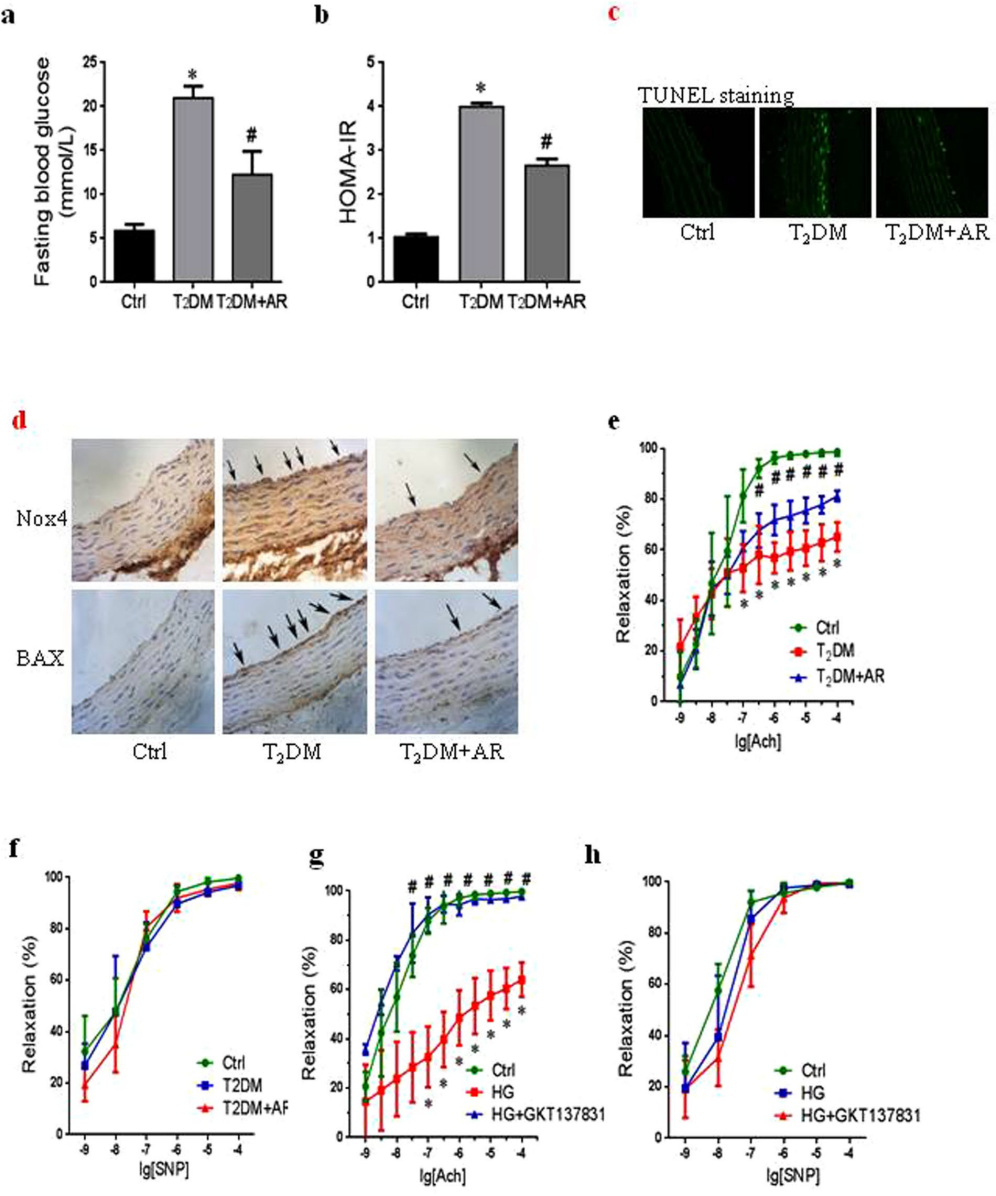
Argirein attenuated vascular dysfunction in T2DM rats. Male Sprague-Dawley rats were fed HFD and a low dose of STZ (40 mg/kg, i.p.) to develop a rat model of type2 diabetes. Diabetic rats were intervened with argirein (AR, 200 mg/kg, ip) for 3 weeks. Summarized data showing the fasting blood glucose (FBG) **(a)** and homeostasis model assessment of insulin resistance (HOMA-IR) **(b)**. Representative fluorescent images of TUNEL assay **(c)** on the aorta showing the TUNEL positive cells on endothelium, and photographs were at 400x magnification (n = 4). Representative immunohistochemistry images showing Nox4 and BAX in the aorta, and photographs were at 100x magnification (n = 4) **(d)**. Vascular relaxant response to acetylcholine (Ach, 10^−10^–10^−4^M) and sodium nitroprusside (SNP,10^−10^–10^−4^M) showing the role of AR on thoracic aorta from diabetic rats **(e**,**f)** and the role of Nox4 inhibitor GKT137831(10^−5^ M, 0.5 h) on aorta rings incubated with HG(50 mM, 3.5 h) (g,h). *P < 0.05 *vs*. Ctrl; ^#^*P* < 0.05 *vs*. T2DM or HG group (n = 6).

Following an injection of STZ in rats with HFD, blood fasting blood glucose (FBG) was significantly elevated relative to control group (Fig. 7a). Simultaneously, homeostasis model assessment of insulin resistance (HOMA-IR) calculated as FBG and serum insulin in T2DM rats significantly increased, compared to control rats (Fig. 7b). Thus, a successful T2DM model was established *in vivo* study. The intervention with argirein in the last 3 weeks resulted in an obvious decrease in FBG and HOMA-IR, which suggested that argirein could attenuate metabolic disorders and systemic insulin resistance in T2DM rats. In addition, the number of TUNEL-positive endothelial cells in argirein-treated group was significantly decreased compared to that in the T2DM rats, which was in line with results *in vitro* (Fig. 7c).

As shown in Fig. 7d, the immunohistochemical analysis revealed no significantly visible precipitation of Nox4 and BAX was observed in the endothelium of aorta in normal rats. However, the definite amount of these proteins could be noted in T2DM rats as indicated by the conspicuous brown granules. The intensified precipitation of Nox4 and BAX proteins was significantly diminished following argirein intervention.

Endothelial cell apoptosis contributes to endothelial function impairment resulting in vascular dysfunction T2DM^3^. Vascular response to acetylcholine and SNP (10^−9^–10^−4^M) was tested, which evoked endothelium-dependent and endothelium-independent vasodilatation in thoracic aorta, respectively (Fig. 7e,f). The endothelium-dependent relaxant responses to acetylcholine were much lower than control rat, which were apparently enhanced in T2DM rat treated with argirein. In the contrast, endothelial-independent vasorelaxation to SNP had no significant difference among three groups showing vascular smooth muscle had the normal ability to relax.

Ultimately, GKT137831, a new type of specific inhibitor of Nox4 was used to explore the regulation of Nox4 on vascular dysfunction^19^. Glucose (50 mM, 3.5 h) incubation with rat thoracic aortas significant impaired the aorta relaxation responding to acetylcholine^20^, suggested that the function of aortic rings was injured *in vitro* (Fig. 7g). GKT137831 treatment showed an effective improvement in aorta relaxation, revealing that the inhibition of Nox4 could reverse endothelium-dependent relaxation suffered from hyperglycemia damage. Similarly, no significant differences on sodium nitroprusside (SNP)-induced relaxation were observed among the control group and high glucose group with or without GKT137831 (Fig. 7h).

## Discussion

In the present study, we provided novel evidences that ET-1/Nox4 signal-dependent O_2_^−.^ generation was critical for suppressing the apoptosis of RAECs triggered by high glucose. Argirein, a derivative of rhein had an anti-apoptosis role through inhibiting the activation of ET-1/Nox4 signal-dependent O_2_^−.^ generation in RAECs. Simultaneously, we demonstrated that T2DM-induced vascular dysfunction was alleviated by argirein intervention through inhibiting Nox4-associated endothelial apoptosis.

Endothelium dysfunction is the hallmark of these vascular complications of diabetes mellitus, and is considered to be the initiating event in atherosclerosis^2^. Apoptosis is the first-identified form of regulated cell death and is considered as the sole form of regulated cell death for decades^21^. Understanding endothelial apoptotic pathways that are altered in vascular complications of diabetes may enable a greater understanding of disease pathogenesis and foster the development of new therapies. Risk factors for cardiovascular diseases including hyperglycemia contributed to vascular endothelial cell dysfunction and apoptosis^9^, which was confirmed in RAECs treated with high glucose in current study. We repeatedly verified that argirein existed an anti-apoptotic role in RAECs as manifested with the normalization of BAX and Bcl-2 and Caspase-3 protein expressions, and the decrease of Annexin V-FITC bound cell percentage and the improvement of DNA ladder in agarose electrophoresis. At the first time, our study provided stronger evidences that argirein was potential in treating vascular complications of diabetes. The previous study from our group found that argirein inhibited hepatocyte apoptosis to blunt hepatosteatosis in diabetic rats^8^. Thus, the anti-apoptotic role of argirein is an underlying mechanism involved in the therapy targeting diabetic vascular complications.

Risk factors for cardiovascular diseases include hyperglycemia and ROS, which collectively contributes to vascular endothelial cell dysfunction. ROS is important mediator of cell growth, adhesion, differentiation, migration, senescence, and apoptosis^22^. Here, we demonstrated that argirein indeed had a apparently anti-oxidative role in RAECs through inhibiting O_2_^−.^ reduction by DHE staining and ESR assay. Argirein exhibited antioxidant activities with a prolonged t_1/2_ compared with rhein, which was also found in diabetic hypogonadism, diabetic nephropathy and diabetic cardiomyopathy^5,13,23^. Rhein was an effective ingredient isolated from Chinese rhubarb (*Rheum Officinale* Baill.) and has been used alone or in a combined therapy^24^. It was reported that rhein protected vascular endothelial cells against oxidative injury through increasing the antioxidant including the SOD and glutathione peroxidase (GSH-PX) activity^25^. However, the solubility of rhein is poor, and its t_1/2_ is not sufficiently long. L-arginine was converted by nitric oxide synthase (NOS) as a donor for NO, which caused sustained suppression of NADPH oxidase-dependent superoxide production in human endothelial cells by S-nitrosylation of p47phox^26^. The chemical modification by connecting a moiety of L-arginine was conducted to improve biological behaviors of argirein^4^. Therefore, the suppression of O_2_^−.^ production was closely associated with its anti-apoptotic role in RAECs. ROS can trigger apoptosis either indirectly through damage to DNA, lipids, and proteins or directly by ROS-mediated activation of signaling molecules. Such proapoptotic signaling by ROS may occur through activation of MAP kinases, such as SAPK/JNK, ERK1/2, and p38^27^. Thus, it is possible that the inactivation of MAP kinases is responsible for anti-apoptosis of argirein.

The major source of ROS generated in the cardiovascular system is the NADPH oxidase, which activation has also been implicated in apoptosis^28^. Argirein markedly attenuated high glucose-induced SOD-sensitive O_2_^−.^ by ESR assay, suggesting that argirein suppressed O_2_^.−^ production dependent on NADPH oxidase. Among other catalytic NADPH oxidase homologues, high expression of Nox4 appears to be a specific characteristic of vascular cells, although Nox2 expression also has been reported^29,30^. In addition, Nox4 upregulation has been implicated in the development of cardiovascular pathologies^31^. Here, we found that Nox4 was responsive for ROS level in RAECs and anti-apoptosis of argirein. Interestingly, there was no significant difference in Nox2 and Nox1 protein expression after the treatment with argirein, which mechanisms still needed further study in future. Thus, Nox4-dependent ROS aggregation is the main cause of vascular endothelial cell apoptosis, the inhibition of which accounted for anti-apoptosis of argirein in RAECs.

ET-1 is a potent vasoconstrictor released mainly from endothelial cells and has been implicated in the development of coronary vascular pathophysiology by acting with ET_A_R and ET_B_R^32^. Hyperglycemia-induced production of ET-1 is a hallmark of endothelial dysfunction in diabetes^33^. ET-1 is able to activate NADPH oxidase in endothelial cells resulting in the disturbance of vascular redox balance^11,12^. Our current study found both ET_A_R and ET_B_R level were increased in respond to high glucose stimulation. However, argirein could only attenuate ET_A_R protein rather than ET_B_R, which was possibly because of the different role of ET_A_R and ET_B_R in vascular injury. ET-1 acting on the ET_A_R, promoted intimal lesion formation following vascular injury^34,35^. ET_B_R mediated NO release and ET-1 clearance in endothelial cells, may moderate lesion formation^36^.Thus, this protective effect of argirein is not mediated by those ET_B_R expressed by endothelial cells. The selective ET_A_R antagonist BQ123 completely prevented high glucose induced elevation of Nox4 expression, which demonstrated that ET-1 induced Nox4 activity through activating ET_A_R and therefore disrupted cell apoptosis induced by high glucose. Thus, ET_A_R /Nox4 constructed a signal pathway which was responsible to anti-apoptotic role of argirein in RAECs. Consistently, our previous study showed that ET_A_R significantly enhanced ROS generation via a NADPH oxidase-dependent pathway implicated in the apoptosis of HK-2 cells^16^.

*In vivo* study, T2DM rats produced by HFD/low STZ impaired endothelium-dependent relaxation in isolated aorta, providing a well‐characterized model of endothelial dysfunction. We found that argirein inhibited systemic insulin resistance and decreased the blood glucose in T2DM rats. The relaxation of aortic rings responding to an endothelium-dependent vasodilator such as acetylcholine but not to an endothelium-independent vasodilator such as SNP was impaired in T2DM rats, whereas the impairment could be prevented by argirein medication. In addition, the impaired endothelium-dependent vasodilation has been established in aorta rings incubated with high glucose condition. Selective Nox4 inhibitor GKT137831 almost completely restored endothelium-dependent relaxation, confirming that argirein mediated vascular relaxation via inhibiting Nox4 activity. Consistent with the results in RACEs, the results from immunohistochemistry suggested that Nox4 and BAX were activated in the aorta of rats fed with HFD/low STZ. Argirein significantly deceased the levels of Nox4 and BAX in the endothelium of aorta, which further confirmed that argirein ameliorated endothelial apoptosis through suppressing Nox4, consequently resulting in relieving the vasodilatation dysfunction. In our recent study, we indeed found that the intervention of argirein was useful to normalize insulin-mediated endothelial dysfunction *via* recovering insulin sensitivity^37^. Here, we didn’t analysis the insulin sensitivity in endothelial cell, but we demonstrated that argirein ameliorated systemic insulin sensitivity in the diabetic rats. Actually, it was well-known that insulin resistance is accompanied by the increases in apoptosis in different cells which is associated with diabetic complication^38,39^. Our previous study also demonstrated that argirein blunted hepatosteatosis through normalizing apoptosis and insulin resistance in the diabetic liver^8^. Thus, it is suggested that the improvement of vascular dysfunction by argirein owed to increasing the insulin sensitivity and inhibiting endothelial cell apoptosis.

In conclusion, we demonstrated that high glucose leaded to the production of ET-1/Nox4 signal-dependent O_2_^−^, consequently triggering the apoptosis in RAECs, which was involved in the pathology of diabetic vascular dysfunction. Argirein can improve vascular function through inhibiting endothelial apoptosis, which was associated with the reduction of ET-1/Nox4 signal-dependent O_2_^−^ production. These findings suggest that argirein is a potential therapeutic drug in alleviating vascular complications in T2DM.

## Electronic supplementary material

Supplementary Information

## References

[1] Akoumianakis, I. & Antoniades, C. Impaired Vascular Redox Signaling in the Vascular Complications of Obesity and Diabetes Mellitus. Antioxidants & redox signaling, 10.1089/ars.2017.7421 (2018). 10.1089/ars.2017.7421 29084432

[2] Prakash K, Chandran DS, Khadgawat R, Jaryal AK, Deepak KK (2016). Correlations between endothelial function in the systemic and cerebral circulation and insulin resistance in type 2 diabetes mellitus. Diabetes & vascular disease research.

[3] Wang Q (2014). Activation of NAD(P)H oxidase by tryptophan-derived 3-hydroxykynurenine accelerates endothelial apoptosis and dysfunction *in vivo*. Circ Res.

[4] Cong XD, Fu PR, Dai DZ, Zhang Y, Dai Y (2012). Pharmacokinetic behavior of argirein, derived from rhein, is characterized as slow release and prolonged T(1)/(2) of rhein in rats. European journal of pharmaceutical sciences: official journal of the European Federation for Pharmaceutical Sciences.

[5] Shi FH (2013). Depressed calcium-handling proteins due to endoplasmic reticulum stress and apoptosis in the diabetic heart are attenuated by argirein. Naunyn-Schmiedeberg’s archives of pharmacology.

[6] Zhang GL (2011). Isoproterenol-induced FKBP12.6/12 downregulation is modulated by ETA and ETB receptors and reversed by argirhein, a derivative of rhein. Acta pharmacologica Sinica.

[7] Yan F (2014). Nox4 and redox signaling mediate TGF-beta-induced endothelial cell apoptosis and phenotypic switch. Cell death & disease.

[8] Shi FH (2013). Hepatosteatosis and hepatic insulin resistance are blunted by argirein, an anti-inflammatory agent, through normalizing endoplasmic reticulum stress and apoptosis in diabetic liver. J Pharm Pharmacol.

[9] Wu N (2016). Acute blood glucose fluctuation enhances rat aorta endothelial cell apoptosis, oxidative stress and pro-inflammatory cytokine expression *in vivo*. Cardiovascular diabetology.

[10] Wang Y (2014). Induction of heme oxygenase-1 ameliorates vascular dysfunction in streptozotocin-induced type 2 diabetic rats. Vascular pharmacology.

[11] Thengchaisri N, Hein TW, Ren Y, Kuo L (2015). Endothelin-1 impairs coronary arteriolar dilation: Role of p38 kinase-mediated superoxide production from NADPH oxidase. Journal of molecular and cellular cardiology.

[12] Li L (2003). Endothelin-1 increases vascular superoxide via endothelin(A)-NADPH oxidase pathway in low-renin hypertension. Circulation.

[13] Hu C (2011). Argirein alleviates diabetic nephropathy through attenuating NADPH oxidase, Cx43, and PERK in renal tissue. Naunyn-Schmiedeberg’s archives of pharmacology.

[14] Frye CA, Patrick CW (2002). Isolation and culture of rat microvascular endothelial cells. In vitro cellular & developmental biology. Animal.

[15] Lui KO (2013). Driving vascular endothelial cell fate of human multipotent Isl1+ heart progenitors with VEGF modified mRNA. Cell research.

[16] Li Q (2016). Inhibition of CPU0213, a Dual Endothelin Receptor Antagonist, on Apoptosis via Nox4-Dependent ROS in HK-2 Cells. Cellular physiology and biochemistry: international journal of experimental cellular physiology, biochemistry, and pharmacology.

[17] Xu M (2012). NAD(P)H oxidase-dependent intracellular and extracellular O2*- production in coronary arterial myocytes from CD38 knockout mice. Free Radic Biol Med.

[18] Wei W, An XR, Jin SJ, Li XX, Xu M (2018). Inhibition of insulin resistance by PGE1 via autophagy-dependent FGF21 pathway in diabetic nephropathy. Scientific reports.

[19] Zhao W (2017). Tert-butyl hydroperoxide (t-BHP) induced apoptosis and necroptosis in endothelial cells: Roles of NOX4 and mitochondrion. Redox biology.

[20] Shuang-Xi W, Li-Ying L, Hu M, Yu-Hui L (2005). Na+/H+ exchanger inhibitor prevented endothelial dysfunction induced by high glucose. Journal of cardiovascular pharmacology.

[21] Peter ME (2011). Programmed cell death: Apoptosis meets necrosis. Nature.

[22] Paniagua Soriano G, De Bruin G, Overkleeft HS, Florea BI (2014). Toward understanding induction of oxidative stress and apoptosis by proteasome inhibitors. Antioxidants & redox signaling.

[23] Xu M (2016). Argirein alleviates stress-induced and diabetic hypogonadism in rats via normalizing testis endothelin receptor A and connexin 43. Acta pharmacologica Sinica.

[24] Zeng CC (2014). The molecular mechanism of rhein in diabetic nephropathy. Evidence-based complementary and alternative medicine: eCAM.

[25] Zhong XF, Huang GD, Luo T, Deng ZY, Hu JN (2012). Protective effect of rhein against oxidative stress-related endothelial cell injury. Molecular medicine reports.

[26] Selemidis S (2008). Suppressing NADPH oxidase-dependent oxidative stress in the vasculature with nitric oxide donors. Clinical and experimental pharmacology & physiology.

[27] Tang K, Li X, Zheng MQ, Rozanski GJ (2011). Role of apoptosis signal-regulating kinase-1-c-Jun NH2-terminal kinase-p38 signaling in voltage-gated K+ channel remodeling of the failing heart: regulation by thioredoxin. Antioxidants & redox signaling.

[28] Leonarduzzi G (2006). Early involvement of ROS overproduction in apoptosis induced by 7-ketocholesterol. Antioxidants & redox signaling.

[29] Van Buul JD, Fernandez-Borja M, Anthony EC, Hordijk PL (2005). Expression and localization of NOX2 and NOX4 in primary human endothelial cells. Antioxidants & redox signaling.

[30] Park HS, Chun JN, Jung HY, Choi C, Bae YS (2006). Role of NADPH oxidase 4 in lipopolysaccharide-induced proinflammatory responses by human aortic endothelial cells. Cardiovasc Res.

[31] Szocs K (2002). Upregulation of Nox-based NAD(P)H oxidases in restenosis after carotid injury. Arterioscler Thromb Vasc Biol.

[32] Gupta RM (2017). A Genetic Variant Associated with Five Vascular Diseases Is a Distal Regulator of Endothelin-1 Gene Expression. Cell.

[33] Hathaway CK (2016). High Elmo1 expression aggravates and low Elmo1 expression prevents diabetic nephropathy. Proc Natl Acad Sci USA.

[34] Chen DD, Dong YG, Yuan H, Chen AF (2012). Endothelin 1 activation of endothelin A receptor/NADPH oxidase pathway and diminished antioxidants critically contribute to endothelial progenitor cell reduction and dysfunction in salt-sensitive hypertension. Hypertension.

[35] Li L (2016). Endothelin Receptor Down-Regulation Mediated Ligand Regulation Mechanisms Protect Against Cellular Hypoxia Injury in Rat Vascular Endothelial Cells. Cellular physiology and biochemistry: international journal of experimental cellular physiology, biochemistry, and pharmacology.

[36] Kirkby NS (2012). Non-endothelial cell endothelin-B receptors limit neointima formation following vascular injury. Cardiovasc Res.

[37] Li Q (2018). Argirein alleviates vascular endothelial insulin resistance through suppressing the activation of Nox4-dependent O2(−) production in diabetic rats. Free Radic Biol Med.

[38] Li S (2017). Excessive Autophagy Activation and Increased Apoptosis Are Associated with Palmitic Acid-Induced Cardiomyocyte Insulin Resistance. Journal of diabetes research.

[39] Liang W (2017). Effects of Taurine and L-Arginine on the Apoptosis of Vascular Smooth Muscle Cells in Insulin Resistance Hypertensive Rats. Advances in experimental medicine and biology.

