# Supplementary material for: Improvement of vascular dysfunction by argirein through inhibiting endothelial cell apoptosis associated with ET-1/Nox4 signal pathway in diabetic rats
Source: Sci Rep. 2018 Aug 22;8:12620. doi: 10.1038/s41598-018-30386-w (PMC6105644; doi:10.1038/s41598-018-30386-w)
Supplement: Supplementary file 1 — Supplementary Information [file 41598_2018_30386_MOESM1_ESM.pdf]

Improvement of vascular dysfunction by argirein through inhibiting endothelial cell apoptosis associated with ET-1/Nox4 signal pathway in diabetic rats

Jie Su, Xing-Rong An, Qing Li, Xiao-Xue Li, Xiao-dong Cong, Ming Xu

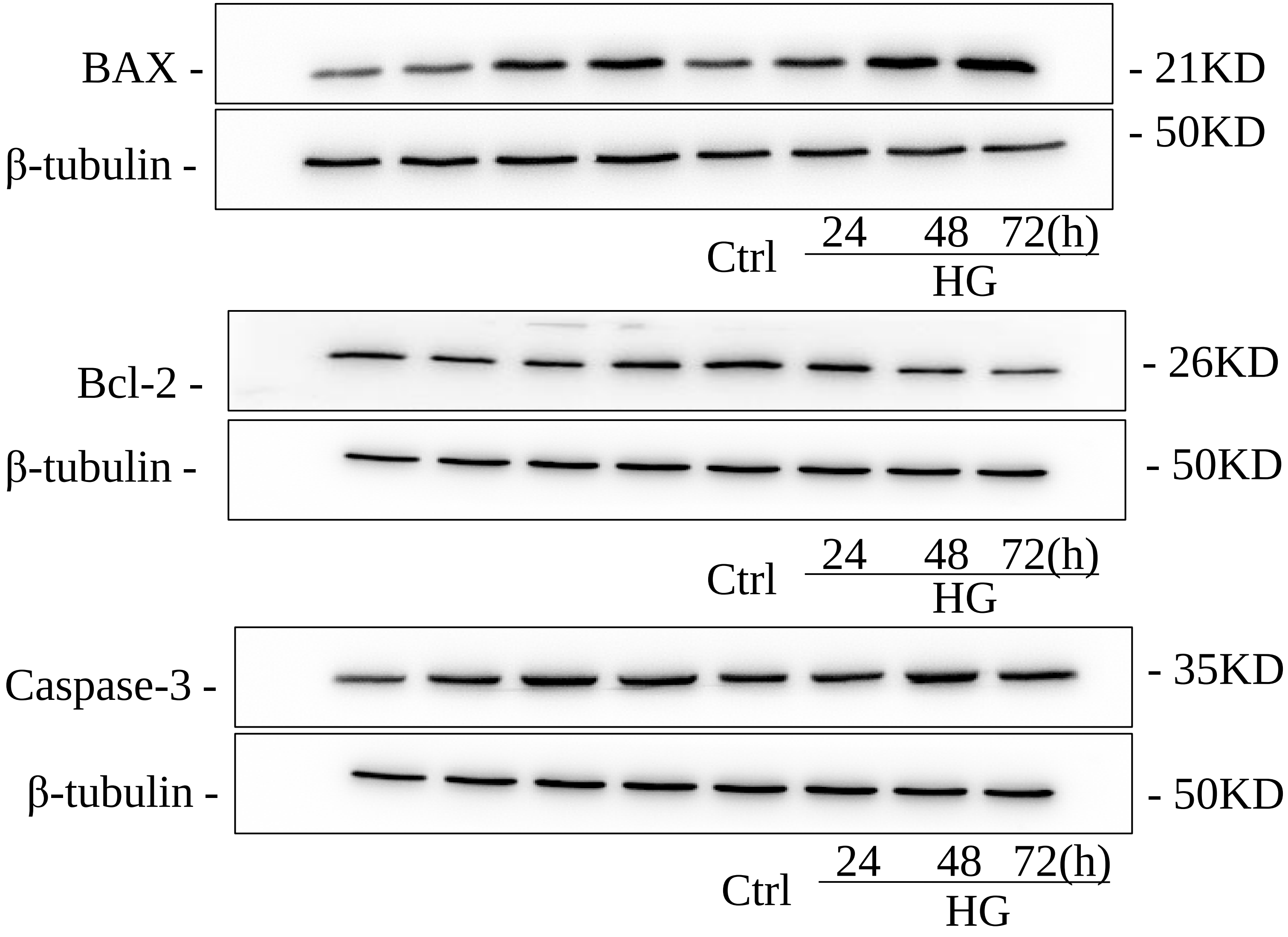
BAX -
- 21KD
β-tubulin -
- 50KD
Ctrl
24
48
72(h)
HG
Bcl-2 -
- 26KD
β-tubulin -
- 50KD
Ctrl
24
48
72(h)
HG
Caspase-3 -
- 35KD
β-tubulin -
- 50KD
Ctrl
24
48
72(h)
HG

Fig.3

a

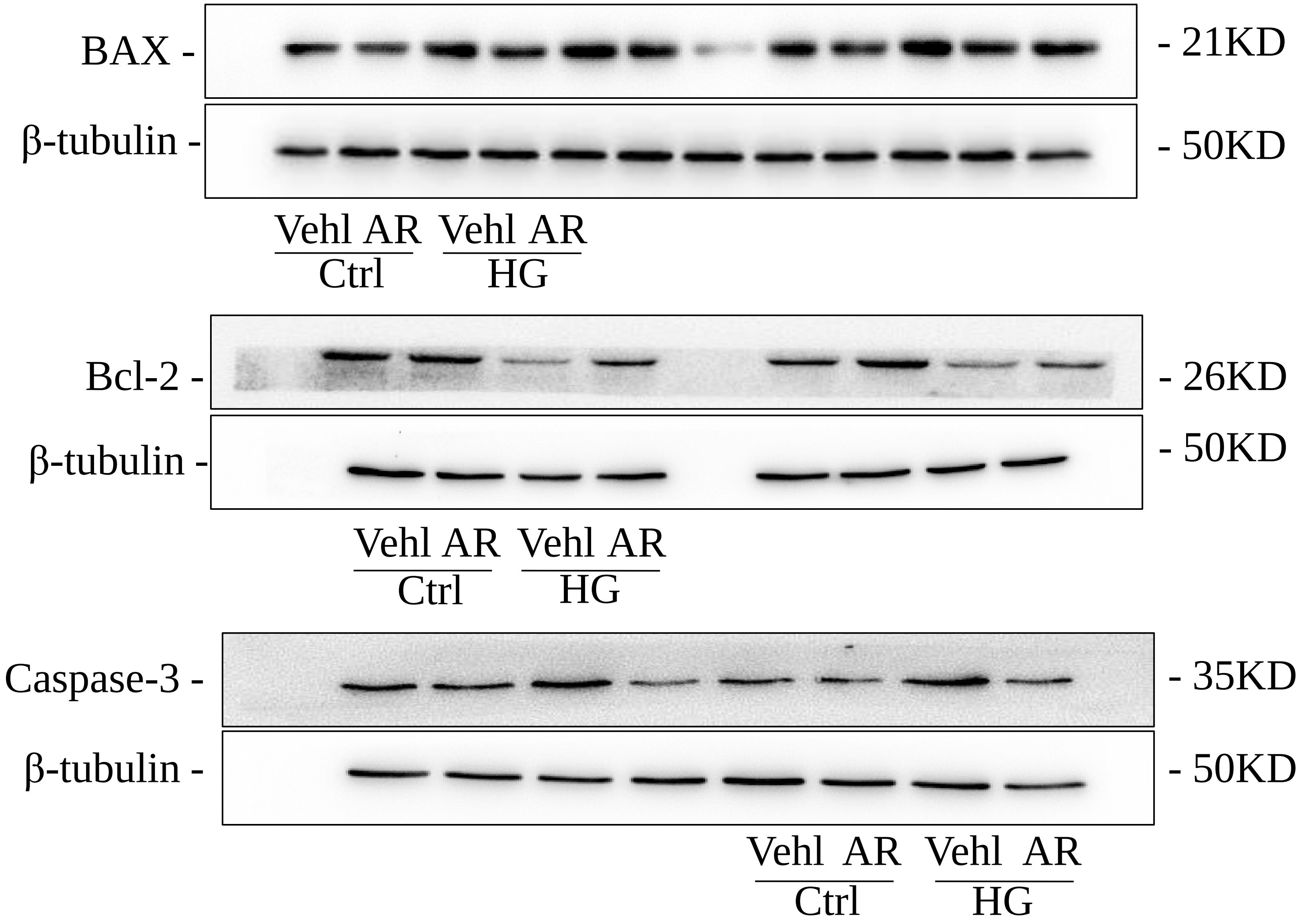
BAX -
- 21KD
β-tubulin -
- 50KD
Vehl AR
Ctrl
Vehl AR
HG
Bcl-2 -
- 26KD
β-tubulin -
- 50KD
Vehl AR
Ctrl
Vehl AR
HG
Caspase-3 -
- 35KD
β-tubulin -
- 50KD
Vehl AR
Ctrl
Vehl AR
HG

Fig.4

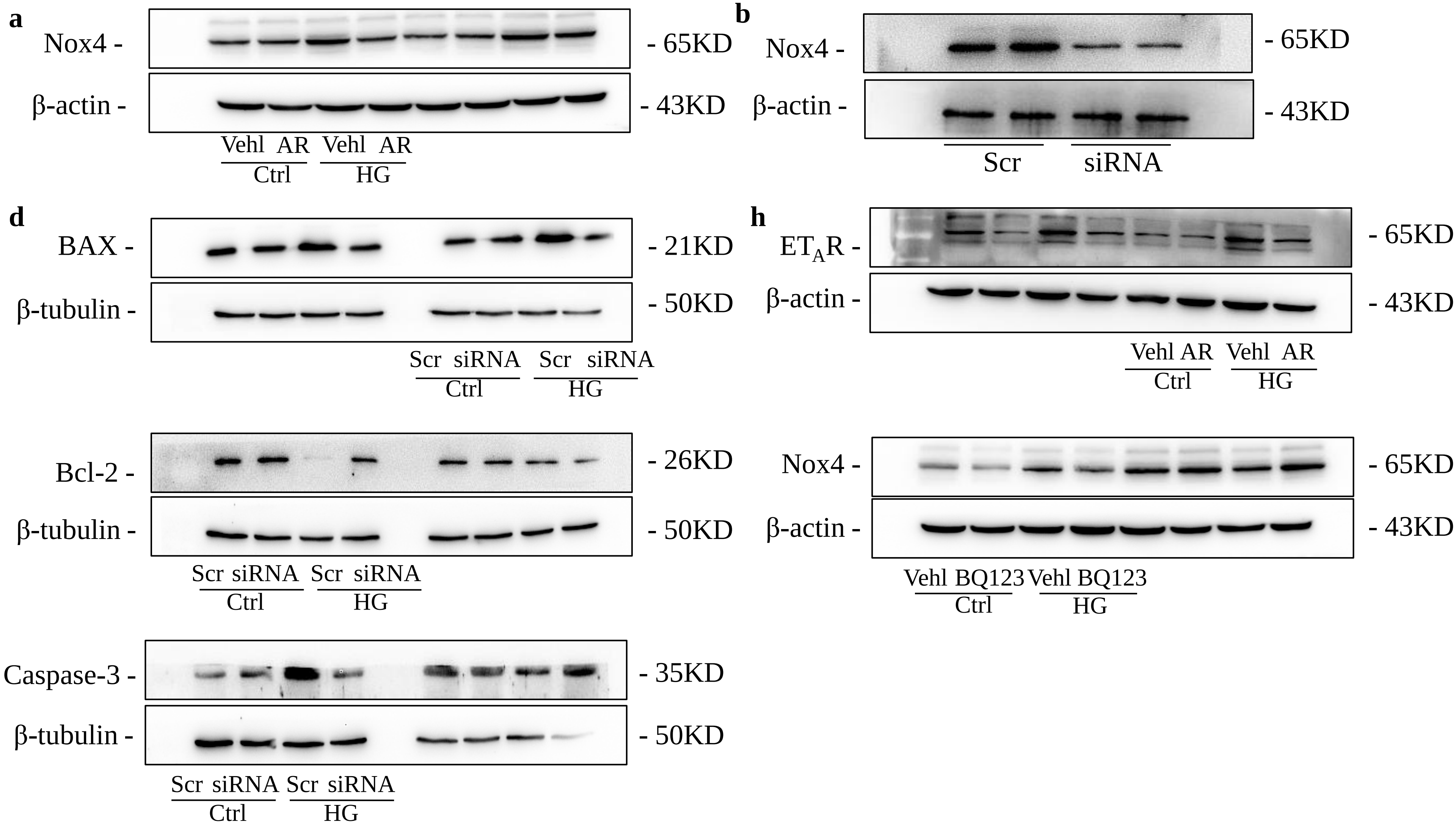
a
Nox4 -
- 65KD
β-actin -
- 43KD
Vehl AR Vehl AR
Ctrl HG
b
Nox4 -
- 65KD
β-actin -
- 43KD
Scr siRNA
d
BAX -
- 21KD
β-tubulin -
- 50KD
Scr siRNA Scr siRNA
Ctrl HG
Bcl-2 -
- 26KD
β-tubulin -
- 50KD
Scr siRNA Scr siRNA
Ctrl HG
Caspase-3 -
- 35KD
β-tubulin -
- 50KD
Scr siRNA Scr siRNA
Ctrl HG
h
ETAR -
- 65KD
β-actin -
- 43KD
Vehl AR Vehl AR
Ctrl HG
Nox4 -
- 65KD
β-actin -
- 43KD
Vehl BQ123 Vehl BQ123
Ctrl HG

Fig.6
